# Complete Genome Sequence of Phytobacter diazotrophicus Strain UAEU22, a Plant Growth-Promoting Bacterium Isolated from the Date Palm Rhizosphere

**DOI:** 10.1128/MRA.00499-20

**Published:** 2020-06-18

**Authors:** Yasmeen Salha, Naganeeswaran Sudalaimuthuasari, Biduth Kundu, Raja S. AlMaskari, AlReem S. Alkaabi, Khaled M. Hazzouri, Synan F. AbuQamar, Khaled A. El-Tarabily, Khaled M. A. Amiri

**Affiliations:** a Department of Biology, College of Science, United Arab Emirates University, Al Ain, United Arab Emirates; b Khalifa Center for Genetic Engineering and Biotechnology, United Arab Emirates University, Al Ain, United Arab Emirates; University of Arizona

## Abstract

Here, we present a complete circular genome (5.4 Mb) and a plasmid (104,972 bp) of the plant growth-promoting bacterium Phytobacter diazotrophicus strain UAEU22, isolated from date palm rhizosphere in the United Arab Emirates (UAE). Annotation of the genome resulted in 5,229 predicted genes.

## ANNOUNCEMENT

Plant growth-promoting rhizobacteria (PGPR) are a group of bacteria which help plants grow. In this study, PGPR Phytobacter diazotrophicus strain UAEU22 was isolated from the rhizosphere of a date palm farm located in the Emirate of Ras Al-Khaimah, United Arab Emirates (UAE) (25.8007°N, 55.9762°E). We followed the same laboratory procedure described in our recent publication (1) for *P. diazotrophicus* isolation from the rhizosphere. A single colony of the *P. diazotrophicus* strain UAEU22 was grown overnight in nutrient broth at 30°C, and DNA from the culture was extracted using an XpressDNA bacterial kit (MagGenome Technologies, Chennai, India) as per the manufacturer’s instructions. The isolated DNA was quantified on a NanoDrop 2000 spectrophotometer (Thermo Fisher Scientific, Waltham, MA) and a Qubit 2.0 fluorometer (Thermo Fisher Scientific). The quality of the DNA was confirmed by the presence of a single compact DNA band using agarose gel electrophoresis. The isolated bacterial genomic DNA was used for 16S rRNA gene-based bacterial typing and whole-genome sequencing (WGS) of the bacterium.

Bacterial typing was carried out by the 16S rRNA gene-based method. PCR primers (forward, 5′-AGAGTTTGATCCTGGCTCAG-3′, and reverse, 5′-GGTTACCTTGTTACGACTT-3′) were used to amplify the 16S rRNA gene. Bidirectional sequencing of the PCR amplicon was performed on a Genetic Analyzer 3500 (Applied Biosystems). In total, 1,418 bp of good-quality (>20) 16S rRNA gene sequences was obtained, and the sequences were searched for homology against the NCBI bacterial database using the online BLAST (2) program. The similarity search confirmed that the generated sequence showed the highest homology with the bacterium Phytobacter diazotrophicus (NCBI accession number NR_115869; >99% identity with an E value of 0.0).

We used both the Oxford Nanopore and Illumina platforms for the WGS of *P. diazotrophicus* strain UAEU22. An Oxford Nanopore MinION-compatible WGS library was prepared with a ligation sequencing kit (SQK-LSK 109), and sequencing was performed on an Oxford Nanopore MinION flow cell (FLO-MIN106D R9.4 revision D chip). Illumina-compatible WGS library preparation was done using a NEBNext Ultra II DNA library preparation kit and sequenced using the Illumina NovaSeq 6000 platform (150-bp paired-end sequencing chemistry).

Guppy v.3.3.2 (implemented in the MinKNOW interface [Oxford Nanopore, Cambridge, UK]) was used for base calling, demultiplexing, and adapter trimming of the MinION-generated reads. In total, we obtained 1,281,904 MinION long reads (1,287,782,011 bp of nucleotides; *N*_50_, 1,300 bp), ranging between 83 and 114,504 bp, which generated an estimated genome coverage of ∼235×. Long-read sequencing errors were corrected using Canu v.1.8 (-correct parameter) (3). After error correction, reads greater than 1,000 bp long were considered for the genome assembly process. For Illumina data, FastQC (4) was used to check the quality of the raw reads, and the Trimmomatic v.0.39 (5) tool was used to trim adapter and low-quality regions found in the reads. In total, 18,605,012 raw reads were obtained from Illumina sequencing (∼900× coverage). After trimming of adapter sequences and low-quality reads, we retained 18,243,330 paired-end reads (98% of reads; quality value, >30), ranging in length between 50 and 150 bp, for further use in the genome assembly.

We used SPAdes v.3.11.1 (6) with default settings for hybrid *de novo* genome assembly (using both Illumina and MinION reads) and genome error correction (using Illumina short reads). BUSCO v.3 (7) was used to confirm the quality and completness of the genome assembly. The NCBI PGAP (8) was used for gene prediction and genome annotation.

The genome assembly of *P. diazotrophicus* strain UAEU22 resulted in a single circular genome of 5,422,265 bp (GC content, 53.2%) and a megaplasmid with a size of 104,972 bp (GC content, 50%). Gene annotation resulted in 5,229 predicted genes (5,119 coding DNA sequences [CDSs], 22 rRNAs, 79 tRNAs, and 9 noncoding RNAs [ncRNAs]) and 62 pseudogenes. We are now carrying out the functional annotation and expression analysis of the predicted genes which are related to nitrogen metabolism.

### Data availability.

The Illumina and Oxford Nanopore reads synthesized during this experiment were deposited in the NCBI SRA database under BioProject accession number PRJNA625126 and BioSample accession numbers SRR11536937 (Illumina PE reads) and SRR11536936 (Oxford Nanopore). The assembled genome and plasmid sequences were submitted to the NCBI GenBank database under accession numbers CP051548 (genome) and CP051549 (plasmid). The bacterial 16S rRNA marker gene sequenced during this study is available at the NCBI GenBank database under accession number MT326197.
